# Expression of odorant‐binding proteins in mouthpart palps of the desert locust *Schistocerca gregaria*

**DOI:** 10.1111/imb.12548

**Published:** 2018-11-27

**Authors:** P. Pregitzer, M. Zielonka, A.‐S. Eichhorn, X. Jiang, J. Krieger, H. Breer

**Affiliations:** ^1^ Institute of Physiology University of Hohenheim Stuttgart Germany; ^2^ Institute of Biology/Zoology, Department of Animal Physiology Martin Luther University Halle‐Wittenberg Halle (Saale) Germany

**Keywords:** insect, desert locust, expression, palps, odorant‐binding protein

## Abstract

Odorant‐binding proteins (OBPs) are essential molecular elements of the insect chemosensory system, which is composed of the antennae and the mouthpart palps (maxillary and labial). In this study, we have analysed the expression and the sensilla specificity of 14 OBP subtypes in the palps of the desert locust *Schistocerca gregaria*. The locust palps comprise only a low number of sensilla basiconica but a high number of sensilla chaetica. Employing a variety of approaches, we found that only a subset of the antennal OBP repertoire was expressed in both palp types. These OBPs were previously shown to be expressed either in sensilla basiconica or sensilla chaetica of the antennae. Comparing the expression pattern in the two chemosensory organs revealed similarities and differences; most remarkably, two OBP subtypes, OBP6 and OBP8, were found in both sensilla types on palps, whereas on the antennae they were solely expressed in one sensillum type. Together, the data indicate a differential, but partly overlapping, expression of OBPs in the two sensilla types of the palps. The differences in the expression pattern of OBP subtypes between antennae and palps might be indicative for distinct functions of the OBPs in the two chemosensory organs.

## Introduction

Chemosensation is of crucial importance to most, if not all, insects, triggering and modulating a variety of essential behaviours. This applies also to the characteristic phase transition of locusts, such as the desert locust *Schistocerca gregaria*, which is dependent on the population density and involves the change from a solitary to a gregarious phase (Hassanali *et al.*, 2005; Simpson and Sword, 2008). This complex transition process requires, besides other stimuli (Roessingh *et al.*, 1998; Simpson *et al.*, 2001; Lester *et al.*, 2005), an interindividual communication mediated by characteristic semiochemicals (Pener and Yerushalmi, 1998; Hassanali *et al.*, 2005; Guo *et al.*, 2011; Wang *et al.*, 2014). The signalling compounds are detected by the two main chemosensory organs: antennae and palps (labial and maxillary). In both organs, the reception of olfactory, gustatory and mechanical stimuli is mediated by morphologically different cuticular hair‐like structures: the sensilla (Blaney, 1977; Ochieng *et al.*, 1998; Jin *et al.*, 2005; 2006). Within the sensillum cavity the dendritic processes of sensory neurons are bathed in aqueous lymph produced by glia‐like supporting cells, which also secrete the characteristic odorant‐binding proteins (OBPs) (Pelosi *et al.*, 2014; 2018). OBPs are small water‐soluble proteins of ~110–200 amino acids, which are supposed to solubilize the mostly lipophilic odorous compounds and may also be involved in signal transduction or termination (Vogt *et al.*, 1999; Sandler *et al.*, 2000; Tegoni *et al.*, 2004; Große‐Wilde *et al.*, 2006; 2007; Leal, 2013; Suh *et al.*, 2014). Analysing the repertoire of potential OBPs of *S. gregaria*, we have identified 14 antennal OBPs from an antennal transcriptome (Jiang *et al.*, 2017), which is a relatively small number compared with the large repertoire (119) of odorant receptors (ORs) (Pregitzer *et al.*, 2017). In the closely related migratory locust *Locusta migratoria*, 16 OBPs have been identified, and 142 ORs were found in genome and antennal transcriptome analysis (Ban *et al.*, 2003; Xu *et al.*, 2009; Yu *et al.*, 2009; Wang *et al.*, 2014; 2015). An evaluation of the topographic expression pattern for the OBP subtypes in the antennae revealed sensilla‐specific and cell‐specific co‐expression of distinct OBP subtypes in *S. gregaria* (Jiang *et al.*, 2017; 2018).

The palps are generally considered to be specialized for the detection of tastants involved in food choice behaviour, and gustatory sensory hairs are highly enriched at the tips of the palps, called ‘domes’. In locusts, the dome region is covered with about 300 hair‐like sensilla, most of them are sensilla chaetica, but some sensilla basiconica have also been identified (Jin *et al.*, 2006). The presence of typical olfactory sensilla basiconica (Blaney and Chapman, 1969a; 1969b, 1970; Jin *et al.*, 2006) suggests that olfactory‐related molecular elements, such as ORs and OBPs, should be expressed in the palps. So far, the knowledge about molecular elements relevant for chemosensation in the palps of locust is limited. Recently, some receptor types and OBPs were found to be expressed in the palps of *L. migratoria* (Wang *et al.*, 2015; Zhang *et al.*, 2017; Li *et al.*, 2018). In this study, we set out to decipher the expression and sensilla specificity of OBPs on labial and maxillary palps in *S. gregaria*. Towards this goal, we have assessed the expression of OBPs in the palps of the desert locust by reverse transcription PCR (RT‐PCR), fluorescence *in situ* hybridization (FISH) experiments on tissue sections and whole‐mount FISH (WM‐FISH).

## Results

### Cellular architecture of the dome region from maxillary and labial palps

**Figure 1:**
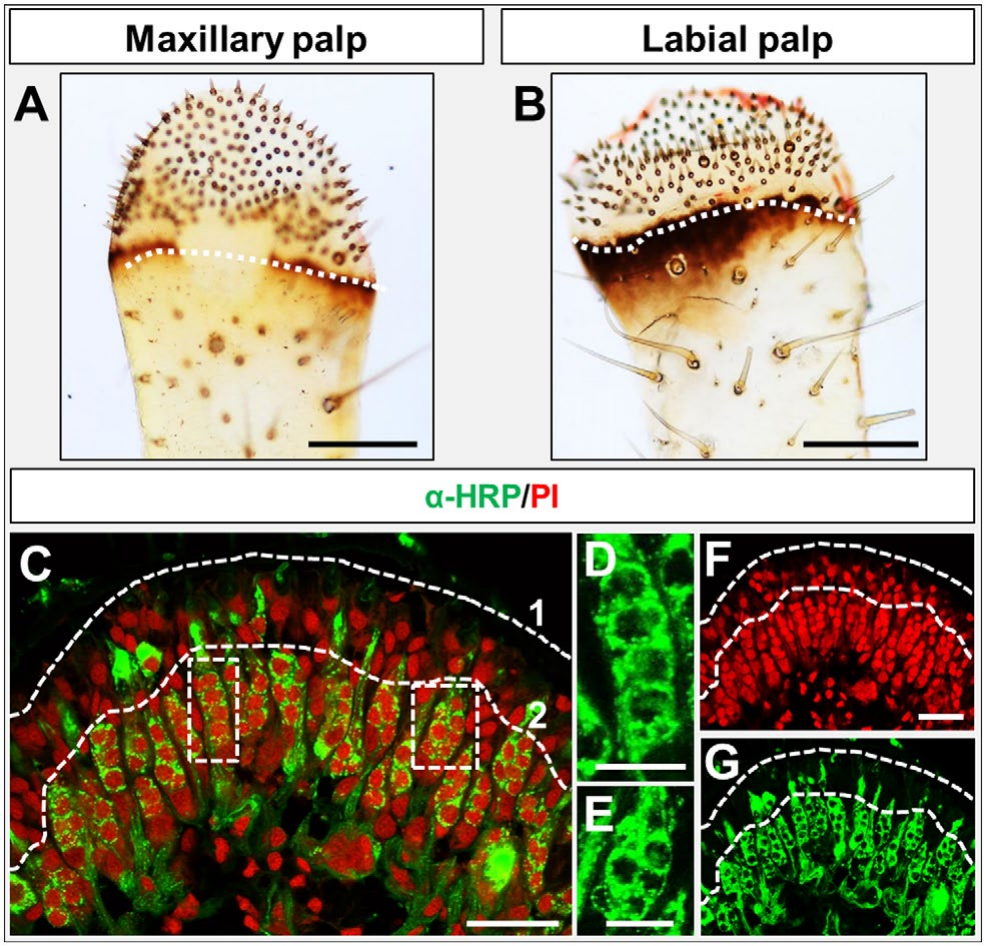
(A, B) Light microscopic images of *Schistocerca gregaria* maxillary and labial palps. The beginning of the dome region is indicated by a white dashed line. The enriched sensilla density on the dome regions is shown. Below the white dashed line different sensilla in lower density are visible. (C) Immunohistochemistry utilizing a horseradish peroxidase (HRP) Alexa‐488 conjugate utilizing a labial palp cryosection combined with propidium iodide nuclei staining. Neuronal α‐HRP‐labelled cells are visualized by green fluorescence; stained nuclei are shown in red fluorescence. Green‐labelled neuronal structures are visible in a palisade fashion. The white dashed line (1) indicates the beginning of the cuticle, whereas below dashed line (2) the HRP‐labelled neurons can be found. (D, E) Magnification of the indicated regions (white dashed line boxes) in (C), showing the ‘longitude’‐type (D) and the ‘round’‐type (E) of labelled cell clusters in single fluorescence channel images (green). (F, G) Single fluorescence channel images of the merged image in (C). Scale bars: A, B, 200 µm;, C, F, G, 50 µm; D, E, 20 µm.

As a first step in the attempt to decipher the expression patterns of OBPs in olfactory and non‐olfactory sensilla of the locust palps, we have visualized the architecture of the appendices using light microscopy (Fig. 1A, B). The analyses were concentrated on the dome region (tip region) of both maxillary and labial palps, which can easily be identified due to the high density of hair‐like sensilla. In order to get a first insight into the cellular architecture we have used the anti‐horseradish peroxidase (α‐HRP) approach (Jan and Jan, 1982) to visualize neuronal cells in the palps. The results indicate that cells labelled by the α‐HRP Alexa 488 conjugate were organized in palisade‐like clusters (Fig. 1C, G). Stained cells were only visible at some distance to the cuticle, and a ‘band’ of nonlabelled cells filled the gap between the labelled cells and the cuticle; nonneuronal cells were also present below and sparsely between the clusters. Upon a detailed inspection, two morphologically different types of nerve cell clusters emerged: a ‘round’ type and a more stretched ‘longitudinal’ type (Fig. 1D, E). No obvious differences between maxillary and labial palps or gender differences were observed in the experiments conducted in this study.

**Figure 2:**
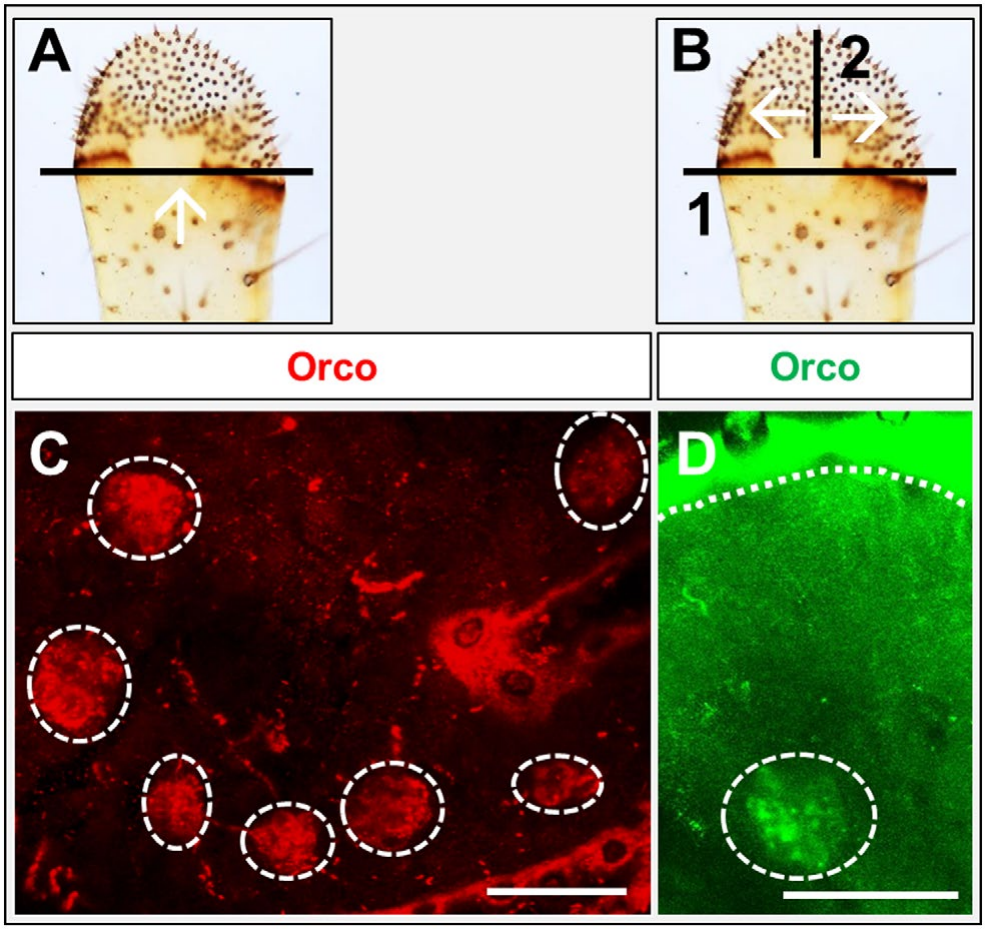
Analysis of whole‐mount fluorescence *in situ* hybridization (WM‐FISH)‐treated palps. (A) As a first step in performing WM‐FISH on palps, a slice closely behind the dome region was performed and images were taken using the confocal laser scanning microscope from the angle indicated by the white arrow. (B) Same as in (A), with the difference that after the first slice at the start (1), at the end of the WM‐FISH procedure a second slice (2) was performed and the images were taken as indicated by the white arrows. (C, D) WM‐FISH utilizing digoxigenin and biotin‐labelled riboprobes of SgreOrco. The image in (C), labial palp, was taken with the angle indicated in (A), whereas the image in (D), maxillary palp, was taken as indicated in (B). The white dash line in (D) indicates the boundary to the cuticle. Images represent projections of optical planes from confocal image stacks. Scale bars: C, D, 50 µm.

The α‐HRP immunohistochemistry (Fig. 1C) did visualize heterogeneous populations of neurons, which in palps may include olfactory, gustatory and mechanosensory neurons. To address the question of whether olfactory sensory neurons (OSNs) are in fact present on palps, maxillary and labial palps were assessed in WM‐FISH experiments for the expression of the olfactory receptor co‐receptor (Orco), a marker for OR‐expressing OSNs. In Fig. 2A, B we indicated the different angles of images taken with the confocal laser scanning microscope in WM‐FISH experiments. The results of a WM‐FISH using an Orco‐specific riboprobe are depicted in Fig. 2C, D. We observed up to 10 clusters of Orco‐positive cells in maxillary as well as in labial palps. All clusters of Orco‐positive cells had a roundish shape, resembling the ‘round’‐type clusters visualized by the α‐HRP‐immunohistochemistry (Fig. 1E). The number and localization of the Orco‐positive cell clusters confirm and extend previous observations regarding the number of olfactory sensilla basiconica present on palps (Blaney and Chapman, 1969b; Jin *et al.*, 2006).

### Expression of OBPs in maxillary and labial palps

**Figure 3:**
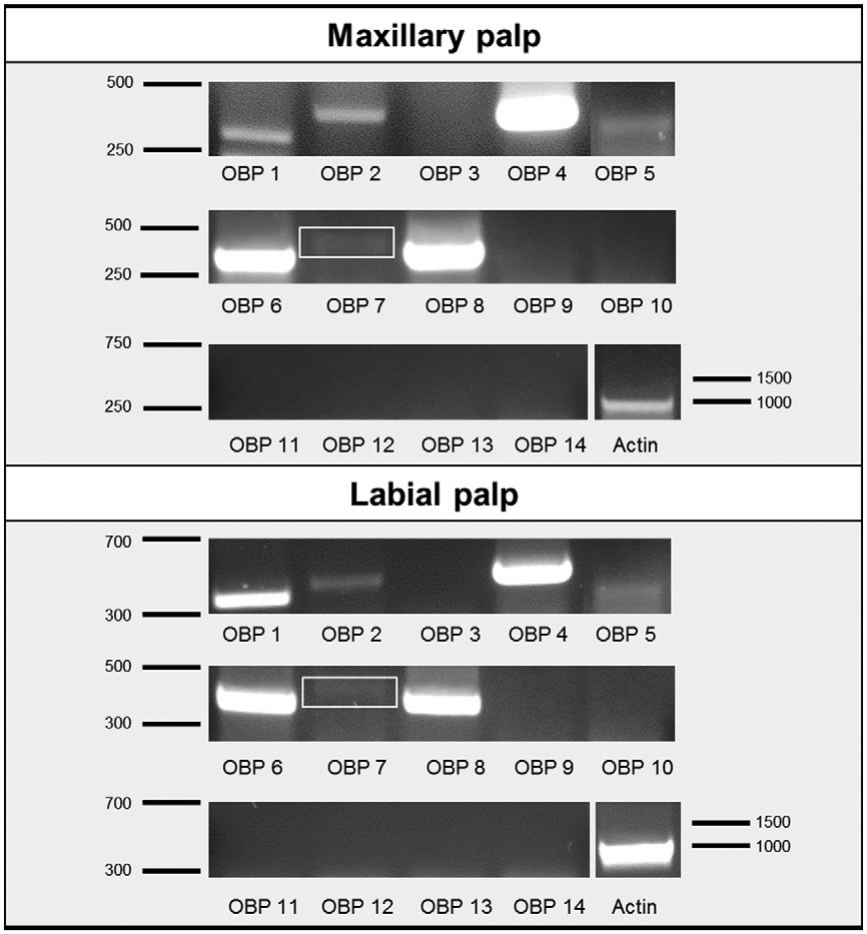
Reverse transcription PCR conducted with cDNA prepared from maxillary palps and labial palps. The respective odorant‐binding protein (OBP) tested is indicated below the lane; actin (AEV89776) was used for control of the cDNA integrity. The numbers right or left of the image indicate marker bands in base pairs. Expression of the OBPs 1, 2, 4, 5, 6, 7 and 8 in both palp types is shown. The white boxes indicate a weak, but visible PCR band for OBP7.

The presence of Orco‐positive cells in the palps leads to the question of what types of OBPs may be expressed in sensilla of the palps. In a previous study we identified 14 antennal OBPs of desert locust *S. gregaria* (Jiang *et al.*, 2017). Specific primers for each of the 14 OBPs were employed in RT‐PCR experiments to assess cDNA from maxillary and labial palps for OBP expression. The results are shown in Fig. 3 and indicate a specific expression of the OBP subtypes 1, 2, 4, 5, 6, 7 and 8 in both palp types. Previously, we found that, in antennae, members of OBP subfamily I‐A (OBP1, OBP5 and OBP6) were expressed in olfactory Orco‐positive sensilla, whereas members of subfamily I‐B (OBP2 and OBP7) as well as members of subfamily III‐A (OBP4) and subfamily III‐B (OBP8) were either expressed in sensilla chaetica or in both sensilla chaetica and sensilla coeloconica (Jiang *et al.*, 2018).

### Subfamily I‐A OBPs

**Figure 4:**
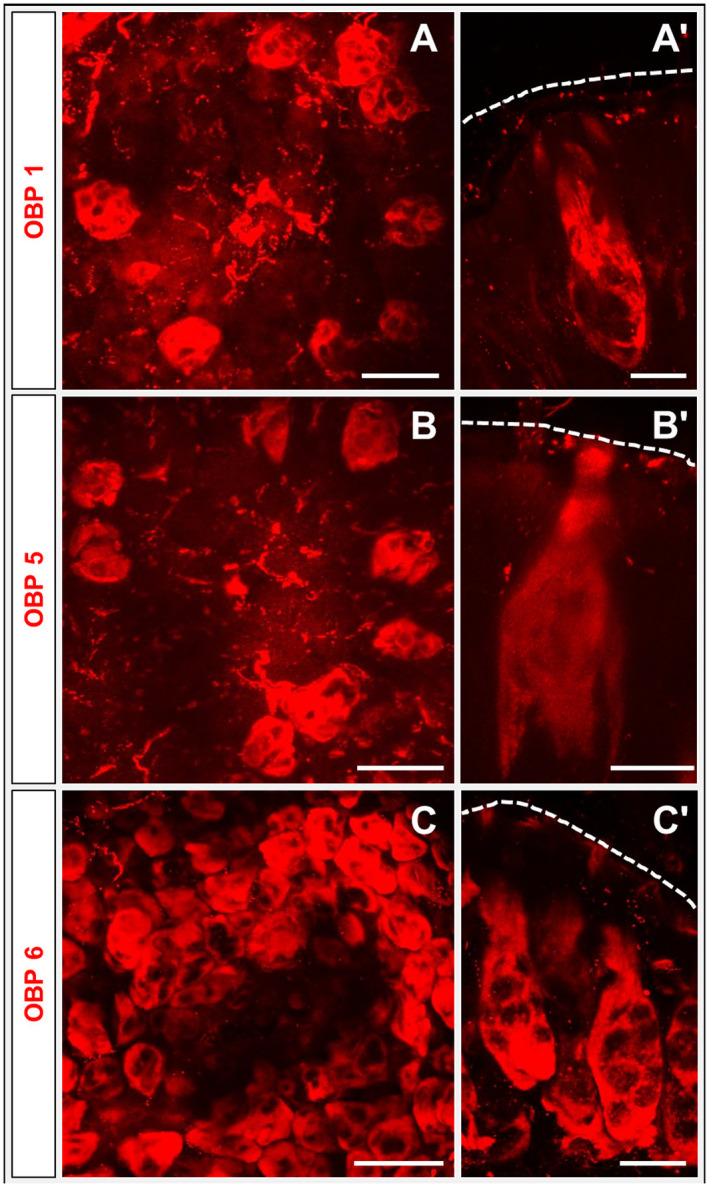
Expression of subfamily I‐A odorant‐binding proteins (OBPs). Whole‐mount fluorescence *in situ* hybridization (WM‐FISH) with labial palps (A–C and A) and maxillary (B and C) utilizing digoxigenin‐labelled riboprobes of subfamily I‐A OBPs: OBP1, OBP5 and OBP6. (A–C) Cells labelled by the probes for OBP1, OBP5 or OBP6 are shown by red fluorescence. The images were taken as indicated in Fig. 2A. (A, B) Expression of OBP1 and OBP5 in a lower number of cell clusters is visible. In contrast, OBP6 is expressed in a higher number of cells as shown in (C). (A–C**)** WM‐FISH utilizing probes for OBP1, OBP5 and OBP6; images were taken as indicated in Fig. 2B. Images represent projections of different optical layers from confocal image stacks. Scale bars: A–C, 50 µm; A–C, 20 µm.

To address the question of whether the three OBPs of subfamily I‐A may be expressed in sensilla basiconica of the palps, similar to their expression pattern in the antennae, WM‐FISH experiments were performed with specific riboprobes for the three OBPs. The results documented in Fig. 4A–C, A–C indicate different labelling patterns for the three subfamily I‐A OBPs. With the probes for OBP1 and OBP5, only a relatively small number of labelled cell clusters were visible (Fig. 4A, B). These results are reminiscent of the small number of Orco‐expressing neuron clusters. Moreover, the longitudinal view, shown in Fig. 4A, B, indicates that the OBP1 and OBP5‐positive cells are located relatively close to the cuticle, and the processes of these cells seem to surround a ‘round’ cluster of OBP‐negative cells. This is even more clearly visible in a video from single plane images of a WM‐FISH using an OBP1‐specific riboprobe (Video S1).

In the case of OBP6 a different labelling pattern emerged: a high number of OBP6‐expressing cells were visualized. Moreover, in contrast to OBP1 and OBP5‐positive cell clusters, the clusters of OBP6‐expressing cells displayed a ‘longitudinal’ shape. In addition, the number of OBP6 cell clusters was much higher than the relatively low number of Orco‐positive cell clusters. The ‘longitudinal’ shape resembles that seen for the majority of cells stained by the α‐HRP immunohistochemistry (Fig. 1C). These observations suggest that OBP1 and OBP5‐positive cells appear to be associated with Orco‐expressing neurons, whereas OBP6‐positive cells are differently distributed. The results were supported by the results of FISH experiments utilizing cryosections demonstrating only low numbers of OBP1 and OBP5‐positive cell clusters and high numbers of OBP6‐labeled cells (Fig. S1).

**Figure 5:**
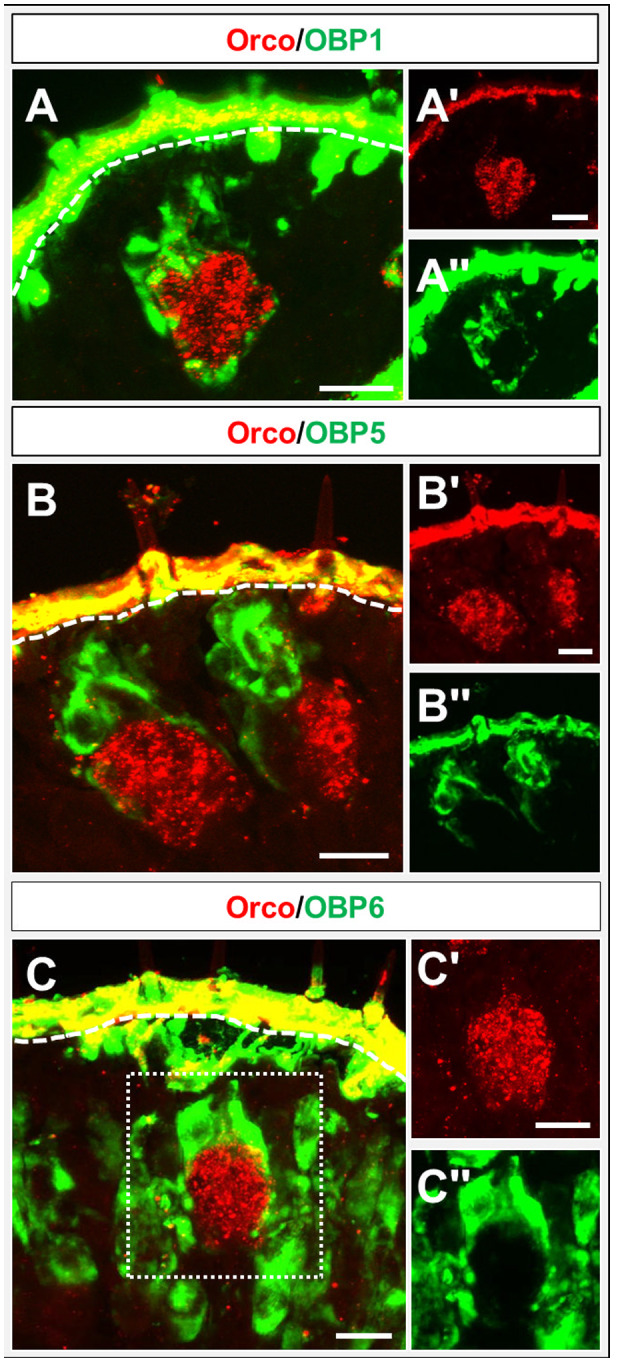
Topography of cells expressing subfamily I‐A OBPs and olfactory receptor co‐receptor (Orco). (A–C) Merged images of fluorescence *in situ* hybridization on sections through the labial (A) and maxillary (B, C) palps of *Schistocerca gregaria* using digoxigenin and biotin‐labelled riboprobes of subfamily I‐A odorant‐binding proteins (OBPs) and Orco. Stained cells are either shown in red (Orco) or green fluorescence (OBPs). A close‐up of labelled OBP‐positive cells (green) and Orco‐expressing cells (red) is shown. The white dashed line indicates the boundary to the cuticle. (A–C, A–C) Single fluorescence channel images; either the green or the red fluorescence are shown. Scale bars: 20 µm.

The notion that OBP1, OBP5 and OBP6 may be expressed in sensilla basiconica was scrutinized in FISH experiments on sections using the olfactory co‐receptor Orco as a marker for olfactory neurons. The results indicate a close association of OBP1 and OBP5‐positive cells with Orco‐expressing cells (Fig. 5A, B). The cell bodies of OBP1 and OBP5‐expressing cells were always above the Orco‐positive neurons; moreover, slight cytoplasmic extensions surrounding the OSN cluster were visible. Interestingly, a close association was also observed for some OBP6‐expressing cells and Orco‐positive OSNs (Fig. 5C). However, the majority of OBP6‐expressing cells were found without any association with Orco‐positive neurons (Fig. 5C).

**Figure 6:**
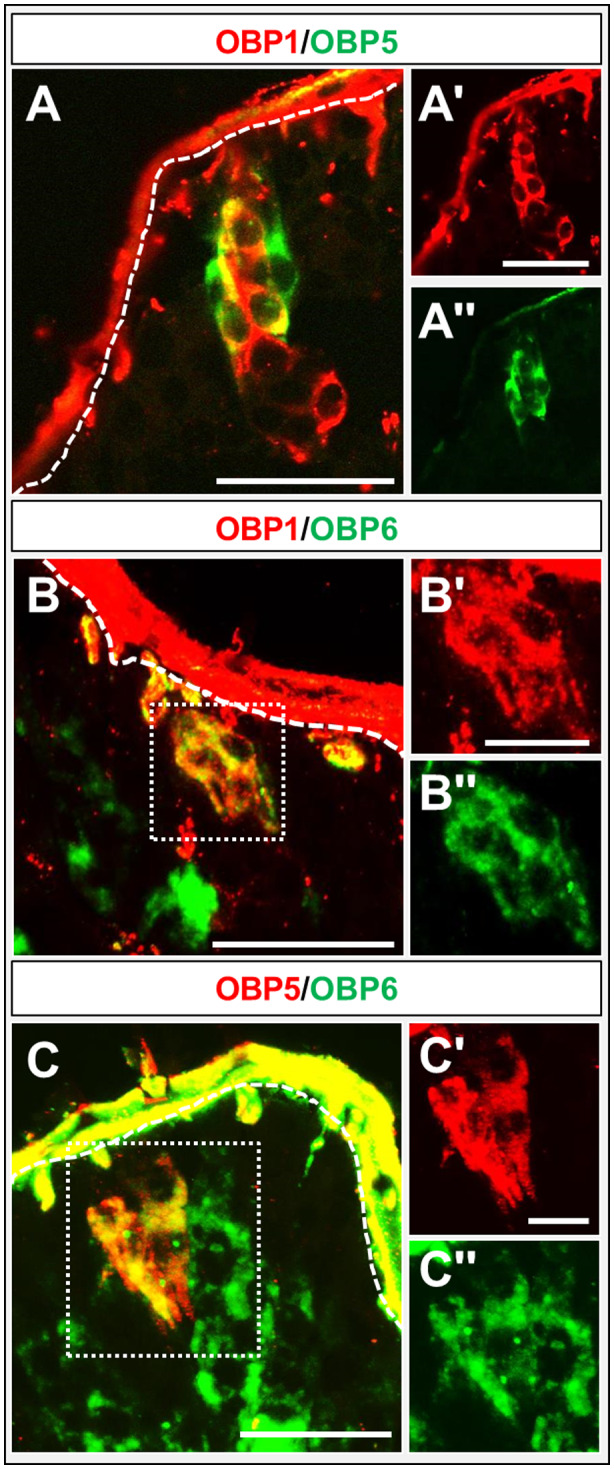
Co‐expression of subfamily I‐A odorant‐binding proteins (OBPs). (A–C) Merged images of fluorescence *in situ* hybridization on sections of maxillary palps of *Schistocerca gregaria* using digoxigenin and biotin‐labelled riboprobes of subfamily I‐A. OBP‐expressing cells are depicted in red and green fluorescence. (A) A partial co‐expression of OBP1 and OBP5 is visible (yellow). (B, C) OBP1 and OBP5‐positive cells co‐express OBP6, indicated by yellow fluorescence. The white dashed line indicates the beginning of the cuticle. (A–C, A–C') Single fluorescence channel images; either the green or the red fluorescence are shown. Scale bars: A–C, A, 50 µm; B, C, 20 µm.

The question of whether OBPs of subfamily I‐A may be co‐expressed by cells in the palps was approached by performing FISH experiments on cryosections from both palp types. The results shown in Fig. 6 indicate that OBP1 and OBP5 are not always co‐expressed (Fig. 6A), but cells expressing OBP5 also expressed OBP1. Cells expressing solely OBP1 were located below the cells co‐expressing OBP1 and OBP5. In the case of OBP6, a general co‐expression with OBP1 and OBP5 was observed (Fig. 6B, C).

### OBP subtypes of subfamily I‐B and subfamily III

**Figure 7:**
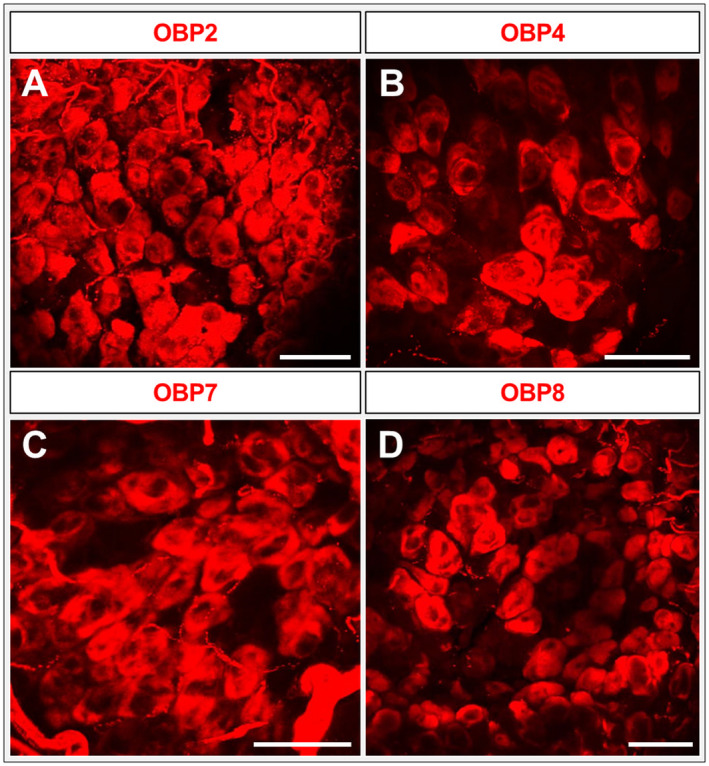
Expression of odorant‐binding proteins (OBPs) from different subfamilies. (A–D) Whole‐mount fluorescence *in situ* hybridization utilizing maxillary (A, B, D) and labial (C) palps of the desert locust using digoxigenin‐labelled riboprobes of subfamily I‐B and subfamily III OBPs (2, 4, 7 and 8). OBPs are visualized by red fluorescence. All four OBPs are expressed in a higher number of cells, similar to OBP6 in Fig. 4. Images represent projections of confocal image stacks representing different optical layers. Scale bars: A–D, 50 µm.

**Figure 8:**
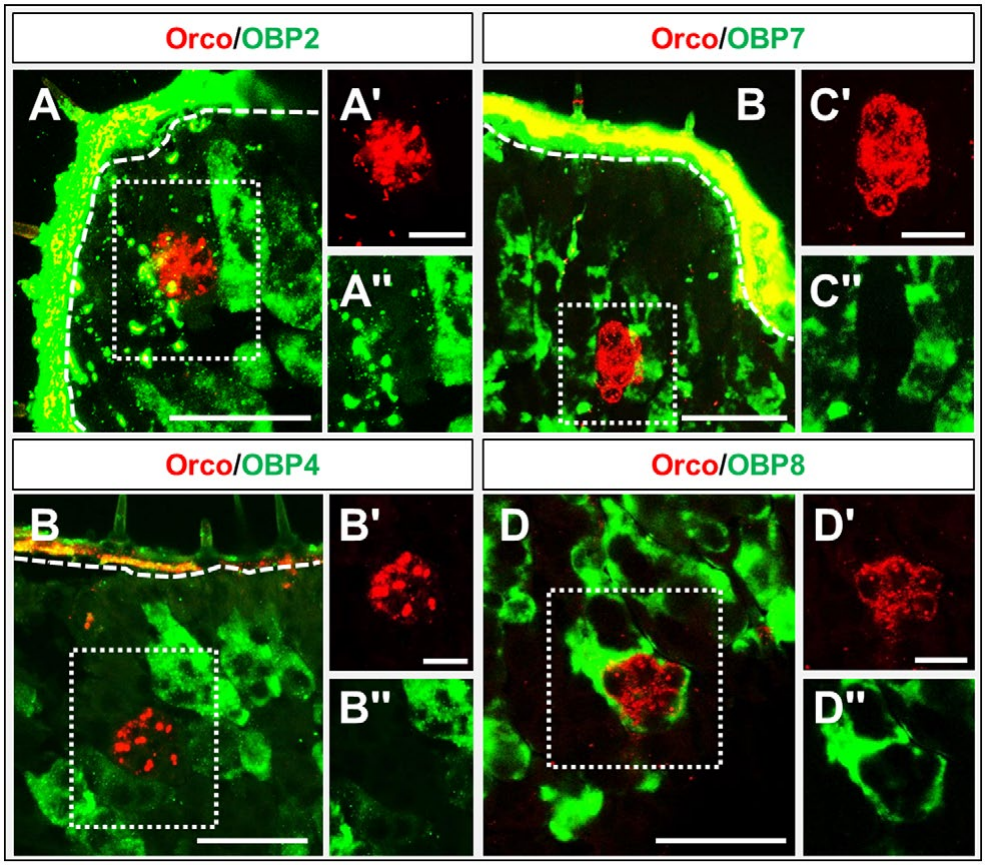
Topography of cells expressing subfamilies I‐B and IV odorant‐binding proteins (OBPs) and olfactory receptor co‐receptor (Orco). (A–D) Merged images from fluorescence *in situ* hybridization experiments on sections of the maxillary (A–C) and labial (D) palps of *Schistocerca gregaria* using digoxigenin and biotin‐labelled riboprobes of subfamily I‐B and subfamily IV OBPs and Orco. OBP‐positive cells are visualized by green fluorescence and Orco‐expressing olfactory sensory neurons (OSNs) by red fluorescence. The OBPs 2, 4 and 7 are not co‐localized with Orco‐expressing OSNs. In contrast, in the case of OBP8, a close‐up of OBP8‐positive cells and Orco‐expressing OSNs is shown. The white dashed line indicates the boundary to the cuticle. (A–D, A–D) Single fluorescence channel images; either the green or the red fluorescence are shown. Scale bars: A–D, 50 µm; A–D, A–D, 20 µm.

**Figure 9:**
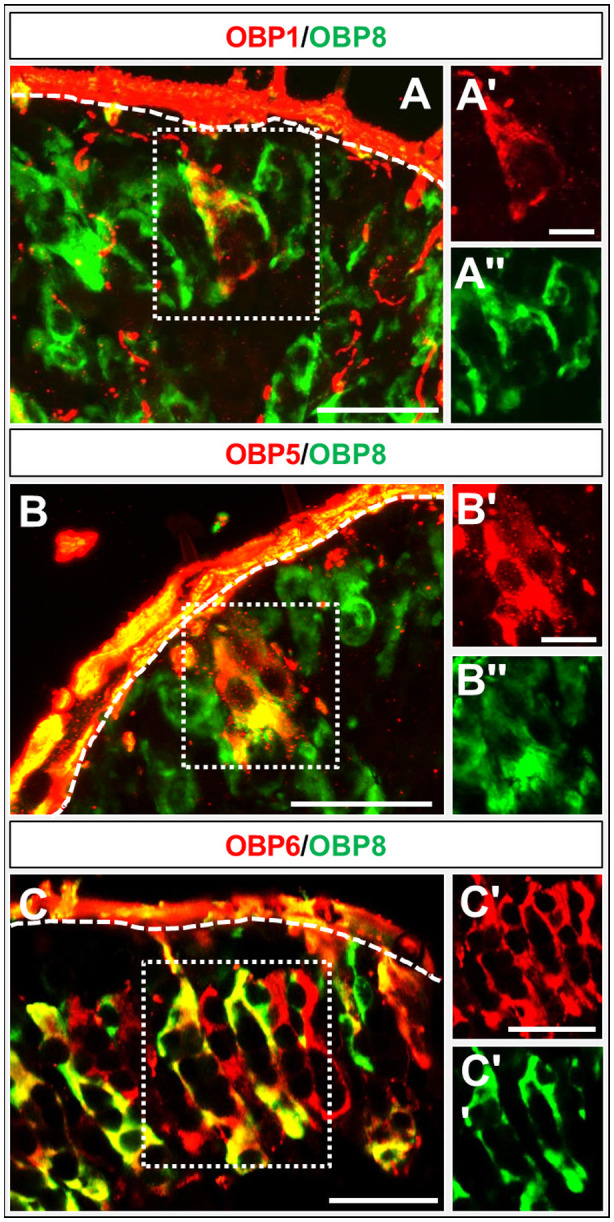
Co‐expression of subfamily I‐A odorant‐binding proteins (OBPs) and OBP8. (A–C) Fluorescence *in situ* hybridization on sections of maxillary (A–C) of *Schistocerca gregaria *with digoxigenin and biotin‐labelled riboprobes of OBP1, OBP5 or OBP6 and OBP8. Family I‐A OBP‐positive cells are depicted by red fluorescence and OBP8‐expressing cells by green fluorescence. Cells expressing OBP1 or OBP5 do co‐express OBP8, indicated by yellow fluorescence. Approximately half of the OBP6‐positive cells co‐express OBP8. The white dashed line indicates the boundary to the cuticle. Images represent projections of different optical layers of confocal image stacks. Scale bars: A–C, A, A, 50 µm; B, C, B, C, 50 µm.

In order to explore the topographic expression of representatives from subfamily I‐B and subfamily III in the palps, we performed WM‐FISH experiments utilizing specific riboprobes for the OBPs 2, 4, 7 and 8 (Fig. 7A–D). For each of the four OBPs, a high number of labelled cells were visible and the labelling pattern was similar to that observed for OBP6 (Fig. 4C). To further evaluate the expression of the four OBPs we also performed FISH experiments on sections (Fig. S2); the results of these experiments were reminiscent of the results obtained by WM‐FISH (Fig. 7). To further specify the topographic expression of these OBPs, FISH experiments on tissue sections were performed using probes for the four OBPs and Orco. In the case of OBP1, OBP5 and OBP6 the OBP‐expressing cells are always above the Orco‐positive OSNs. Moreover, labelled cytoplasmic extensions also surround the Orco‐expressing cells. The results shown in Fig. 8 did show that no such features can be observed regarding cells expressing OBP2, OBP4 or OBP7. However, some cells expressing OBP8 were clearly associated with Orco‐positive OSNs. Therefore, the question arises as to whether OBP8 may be co‐expressed with OBPs of subfamily I‐A. The results of the appropriate co‐labelling experiments (Fig. 9) indicated that OBP1 and OBP5 were always co‐expressed with OBP8 (Fig. 9A, B); of course, there were a high number of cells expressing only OBP8. In the case of OBP8 and OBP6, approximately half of the cells expressing OBP6 also expressed OBP8 (Fig. 9C). The results regarding the sensilla‐specific expression are summarized in Table 1. In this study we identified seven OBPs (1, 2, 4, 5, 6, 7 and 8) to be expressed in palps: two are specifically expressed in sensilla basiconica (OBP1 and OBP5), three in sensilla chaetica (OBP2, OBP4 and OBP7) and, surprisingly, two are expressed in both sensilla types (OBP6 and OBP8).

**Table 1:** Expression of odorant‐binding proteins (OBPs) in different sensilla of the antenna and palps

	Antenna		Palps		
	Sensilla basiconica	Sensilla chaetica	Sensilla basiconica		Sensilla chaetica
OBP1	✓		✓		
OBP5	✓		✓		
OBP6	✓		✓	✓	
OBP2		✓		✓	
OBP4		✓		✓	
OBP7		✓		✓	
OBP8		✓	✓	✓	

Overview of the expression of OBPs in either sensilla basiconica and/or sensilla chaetica of the antennae (Jiang *et al.*, 2017; 2018) or palps of *Schistocerca gregaria*. The expression pattern was similar in labial and maxillary palps.

## Discussion

Insects have developed a highly sophisticated sense of smell based on a variety of specific molecular elements, including OBPs and ORs. OBPs are supposed to be involved in solubilizing and transferring the hydrophobic odorants, but may have additional functions as well (Pelosi, 1994; Xu *et al.*, 2005; Pelosi *et al.*, 2014; 2018). Although most of our knowledge about OBPs is based on studies concentrating on holometabolous dipteran and lepidopteran species (Vogt *et al.*, 1991; Robertson *et al.*, 1999; Harada *et al.*, 2008; Schultze *et al.*, 2013; Pelosi *et al.*, 2014), progress has also recently been made in analysing OBPs of hemimetabolous locusts, notably the desert locust *S. gregaria *and the migratory locust *L. migratoria *(Jin *et al.*, 2005; Xu *et al.*, 2013; Zhang *et al.*, 2013; Wang *et al.*, 2014; 2015; Zhang *et al.*, 2015; Jiang *et al.*, 2017; 2018; Pregitzer *et al.*, 2017; Zhang *et al.*, 2017; Li *et al.*, 2018).

For the desert locust, 14 OBPs have been identified and were found to be expressed in the primary chemosensory organ: the antennae (Jiang *et al.*, 2017). In addition, locusts also utilize their mouthpart palps, maxillary and labial, for chemosensory reception, and relevant sensilla types have previously been described for the palps of *L. migratoria *(Jin *et al.*, 2006). The notion that OBPs may also be expressed in chemosensory sensilla of the palps of *S. gregaria* was scrutinized by RT‐PCR experiments, and the results indicate that, in fact, a subset of the antennal OBPs was also expressed in the palps (Fig. 2). Interestingly, the antennal OBP subtypes were expressed either in sensilla basiconica (OBP1, OBP5 and OBP6) or in sensilla chaetica (OBP2, OBP4, OBP7 and OBP8) (Jiang *et al.*, 2017; 2018). OBPs expressed in sensilla coeloconica of the antenna, like members of OBP subfamily II‐A (Jiang *et al.*, 2017), were not found to be expressed in the palps. These results are in line with the finding that only sensilla basiconica and sensilla chaetica are present on the dome (tip region) of the palps (Jin *et al.*, 2006).

Utilizing the olfactory co‐receptor Orco as marker for OSNs, Orco‐positive cells were visualized in the palps of *S. gregaria*; these putative OSNs were organized in clusters. This observation and recent findings for the closely related locust *L. migratoria* (Zhang *et al.*, 2017; Li *et al.*, 2018) suggest that the palps indeed have a functional role for odour detection.

Previously, it was suggested that OSNs in the palps may be tuned to the detection of distinct odorants and contribute to feeding behaviour, including a short‐range food selection (Blaney, 1974; 1977; Blaney and Chapman, 1969a, 1969b, 1970; Blaney and Duckett, 1975; Haskell and Mordue (Luntz), 1969; Mordue Luntz, 1979). In fact, a recent study provided strong evidence that OBPs and ORs in the palps are involved in detecting specific odours during the feeding process of locust (Li *et al.*, 2018). The recent discovery that SNMP1, a marker for pheromone‐sensitive neurons, is also expressed in the palps points to the possibility that some OSNs in the palps may even be responsive for pheromones (Jiang *et al.*, 2016.

Double labelling experiments have shown that OBP1 and OBP5, members of the OBP subfamily I‐A, were expressed in a low number of cell clusters and in close proximity to Orco‐positive cells (Figs 3 and 4). This result implies that OBP1 and OBP5 are expressed in sensilla types housing OSNs, similar to the expression pattern found on the antennae (Jiang *et al.*, 2017). In addition, the co‐expression patterns seem to be conserved between antennae and palps. OBP5 was always found to be co‐expressed with OBP1, but only a subpopulation of OBP1‐positive cells also expressed OBP5 (Fig. 5). In contrast, a high number of cells expressing OBP6 were identified in the palps, indicating an expression in sensilla chaetica. Yet, a subset of OBP6‐positive cells could be located in close vicinity to Orco‐expressing OSNs. These results indicate that OBP6 is expressed in both sensilla types of the palpal dome. Its broader expression pattern in the palps may imply that OBP6 could be involved in both olfaction and gustation, whereas the expression pattern on the antennae suggests a contribution in olfaction only. This finding may be considered as a first hint that, in the palps, olfactory and gustatory reception may work in concert, a notion that is supported by the finding that, in the antennae, OBP8 was exclusively expressed in sensilla chaetica (Jiang *et al.*, 2018) but in the palps OBP8 was found to be expressed in both sensilla types (Fig. 7).

It is noteworthy that the number of OBP subtypes expressed in sensilla basiconica is similar for both antennae and palps: OBPs 1, 5, 6 and 9 in the antennae and OBPs 1, 5, 6 and 8 in the palps. Remarkably, OBP9, which is expressed in all four sensilla types in the antennae (Jiang *et al.*, 2018), was not expressed in the palps.

The expression of OBPs in sensilla chaetica (Figs 6 and 7) suggests that these subtypes may be involved in gustation and/or mechanosensation. An expression of OBPs in gustatory sensilla has previously been reported in the vinegar fly *Drosophila melanogaster* (Jeong *et al.*, 2013). The broad and strong expression of OBPs in sensilla chaetica might be related to the structure of this sensilla type in locust, which is composed of two compartments: an inner and an outer space (Ochieng *et al.*, 1998; Shanbhag *et al.*, 2001; Zhou *et al.*, 2009). The only study on this issue in *L. migratoria* provided evidence for OBPs in the outer compartment, which is puzzling since sensory dendrites are supposed to be restricted to the inner compartment (Yu *et al.*, 2009). The question as to whether *S. gregaria *OBP subtypes are present in the outer or inner compartment remains to be explored.

The number of OBP subtypes expressed in the palps was smaller than in the antennae. Generally, in locusts the number of OBP subtypes is rather low relative to the number of ORs and compared with other insect species, where the number of OBP subtypes and OR subtypes is often quite similar (Clyne *et al.*, 1999; Vosshall *et al.*, 1999; Hekmat‐Scafe *et al.*, 2002; Justice *et al.*, 2003; Xu *et al.*, 2003; Biessmann *et al.*, 2005). For *Drosophila* antennae, distinct expression patterns of OR and OBP subtypes have been described, indicating that certain ORs are strictly co‐localized with certain OBPs in defined sensilla types (Galindo and Smith, 2001; Shanbhag *et al.*, 2001; Larter *et al.*, 2016). In locusts, currently only four OBPs seem to be expressed in sensilla basiconica (and sensilla trichodea), whereas the number of OR subtypes is much higher. This seems to be reminiscent of the olfactory system of rodents, with over 1000 OR types but a limited number of OBP subtypes (Pevsner *et al.*, 1988a, 1988b; Tegoni *et al.*, 2000; Zhang and Firestein, 2002). Consequently, it has been suggested that the binding spectrum of mouse OBP subtypes is rather unspecific. In locusts, a similar scenario emerges notably for sensilla basiconica, which accommodate a large number of neurons in each sensillum, where a high number of OR subtypes but only a low number of OBP subtypes are expressed.

## Experimental procedures

### Animals and tissues treatment

Adult *S. gregaria* were purchased from local suppliers. The labial and maxillary palps were dissected using autoclaved surgical scissors. For RNA extraction, the organs were immediately frozen in liquid nitrogen and stored at −70 ℃. For *in situ *hybridization experiments on tissue sections the palps were embedded in Tissue‐Tek O.C.T. compound (Sakura Finetek, Alphen aan den Rijn, Netherlands). In the case of whole‐mount *in situ* hybridization, palps were prepared and directly transferred into fixation solution.

### RT‐PCR

Total RNA was extracted from maxillary and labial palps of adult males and females using Extrazol reagent (Blirt, Gdańsk, Poland) following the manufacturer’s protocol. Poly(A)^+^ RNA was isolated and converted into cDNA as described earlier (Pregitzer *et al.*, 2017). Nonquantitative RT‐PCR utilizing SgreOBP‐specific sense and antisense primers (for primers, see Jiang *et al.*, 2017; 2018) were performed with the following conditions: 94 ℃ for 90 s followed by 20 cycles with 94 ℃ for 30 s, 50–60 ℃ for 30 s (thereby, the annealing temperature was decreased by 0.5 ℃ per cycle) and 72 ℃ for 90 s. Subsequently, 20 cycles with an annealing temperature of 40–50 ℃ were performed followed by incubation at 72 ℃ for 15 min. Alternatively, PCR reactions were run at 97 ℃ for 1 min followed by 34 cycles with 97 ℃ for 40 s and 68 ℃ for 3 min. After the last cycle, a final incubation step at 68 ℃ for 3 min was performed. PCR products were run on 1% agarose gels and visualized by staining with ethidium bromide.

### *In situ* hybridization on sections and whole‐mount *in situ* hybridization

Digoxigenin (DIG)‐labelled or biotin‐labelled antisense probes were generated as described by Jiang *et al.* (2017; 2018). Two‐colour FISH on tissue sections was performed as described earlier (Jiang *et al.*, 2017; Pregitzer *et al.*, 2017). WM‐FISH was performed with freshly prepared labial and maxillary palps, which were directly transferred into fixation solution [4% paraformaldehyde in 0.1 M sodium bicarbonate (NaHCO_3_), pH 9.5] and incubated overnight (12–14 h) at 4 ℃. Thin‐walled PCR tubes (0.25 ml; Kisker Biotech, Steinfurt, Germany) were used for all incubation and washing steps with a slow rotation or moderate shaking of tubes at room temperature. After fixation, palps were washed for 1 min in phosphate‐buffered saline [PBS: 0.85% sodium chloride (NaCl), 1.4 mM potassium dihydrogen phosphate, 8 mM sodium hydrogen phosphate, pH 7.1], 0.03% Triton X‐100 and then incubated for 10 min in 0.2 M hydrochloric acid, 0.03% Triton X‐100 and washed twice in PBS for 2 min each. Subsequently, palps were incubated in acetylation solution (0.25% acetic anhydride freshly added in 0.1 M triethanolamine) for 10 min. Afterwards, palps were washed three times in pheromone binding proteins, 0.03% for 5 min. Prehybridizing was conducted using *in situ *hybridization solution (50% formamide, 5 × saline sodium citrate, 1 × Denhardt’s reagent, 50 μg/ml yeast RNA, 1% Tween 20, 0.1% CHAPS, 5 mM ethylenediaminetetraacetic acid, pH 8.0) for 30 min at 4 ℃. The solution was then replaced by hybridization solution containing the respective labelled antisense RNA probes and incubated at 60 ℃ for 72 h. Post hybridization the palps were washed for 4 × 15 min each in 0.1 × saline sodium citrate, 0.03% Triton X‐100 at 60 ℃ and subsequently incubated in 1% blocking reagent (Roche, Indianapolis, IN, USA) in TBS (100 mM tris(hydroxymethyl)aminomethane, pH 7.5, 150 mM NaCl), 0.03% Triton X‐100 for at least 5 h at 4 ℃. DIG‐labelled antisense RNA probes were detected by using an anti‐DIG alkaline‐phosphatase‐conjugated antibody (Roche) diluted 1 : 500 in TBS, 0.03% Triton X‐100, 1% blocking reagent; biotin‐labelled probes were detected by employing a streptavidin horse radish peroxidase conjugate (1 : 100, TSA kit; Perkin Elmer, Boston, MA, USA). After incubation at 4 ℃ for 72 h, palps were washed five times in TBS with 0.05% Tween 20 for 10 min each. Visualization of DIG‐labelled riboprobes was done using the HNPP/Fast Red TR (Roche) substrate diluted 1 : 100 in DAP buffer (100 mM tris(hydroxymethyl)aminomethane, pH 8.0, 100 mM NaCl, 50 mM magnesium chloride) and incubated for 5–7 h at 4 ℃ and washed 3 × 5 min in TBS with 0.05% Tween 20. In the case of double *in situ *hybridizations with a biotin‐labelled riboprobe, after HNPP development the palps were treated with the components of the TSA Fluorescein System (Perkin Elmer) overnight at 4 ℃. Finally, palps were washed 3 × 5 min each in TBS with 0.05% Tween 20 and briefly rinsed in PBS before they were mounted in Mowiol^®^ (10% polyvinyl alcohol 4–88, 20% glycerol in PBS).

### Fluorescence immunohistochemistry

Fluorescence immunohistochemistry was performed on 14 µm cryosections (obtained with a Leica CM3050S cryostat) of *S. gregaria* adult male and female labial and maxillary palps. Sections were transferred to fixation solution (4% paraformaldehyde in 0.1 M NaHCO_3_, pH 9.5) for 22 min at 4 ℃. Afterwards, they were washed three times in PBS for 5 min each at room temperature, followed by an incubation step with blocking solution (10% normal goat serum in PBS) for 1 h at 4 ℃. Blocking solution was replaced by blocking solution containing Alexa Fluor 488‐conjugated goat anti‐horseradish peroxidase antibodies 1 : 500 (Jackson ImmunoResearch, West Grove, PA, USA) and incubated overnight at 4 ℃. The following day the sections were washed 3 × 5 min each in pheromone binding proteins at room temperature, followed by an incubation for 1 h with PBS containing propidium iodide (1 : 1000) for nuclei staining. Finally, the sections were washed again 3 × 5 min each in PBS at room temperature and mounted in Mowiol.

### Analysis of antennal sections by confocal microscopy

Palps whole‐mount preparations and sections probed by WM‐FISH, FISH and fluorescence immunohistochemistry experiments were analysed on a Zeiss LSM 510 meta laser scanning microscope (Zeiss, Oberkochen, Germany). Confocal image stacks of the red and green fluorescence channels and the transmitted‐light channel were taken. Image stacks were utilized to generate pictures showing projections or selected optical planes, as well as video sequences, with the fluorescence and transmitted light channels overlaid or shown separately.

## Supporting information


**Figure S1** **.** (A–C) FISH on section of labial (A and B) and maxillary (C) palps using biotin‐labeled riboprobes of subfamily‐IA OBP1, OBP5 and OBP6. The white dash‐line indicates the boundary to the cuticle. (A′–C′) Magnification of the boxed area in A‐B (white dash‐line). Images represent projections of different optical layers from confocal image stacks or are a single confocal image from an image stack. Scale bars: A–C 50 μm, A′ 20 μM and B and C′ 10 μm.
**Figure S2** **.** (A–C) FISH on maxillary (A, B and D) and labial (C) palps of *Schistocerca gregaria *with digoxigenin‐ and biotin‐labeled riboprobes of OBP2, OBP4, OBP7 and OBP8. The white dash‐line indicates the boundary to the cuticle. Images represent projections of different optical layers from confocal image stacks or are a single confocal image from an image stack. Scale bars: A–D 50 μm.

**Video S1** **.** A video generated from single plane confocal images of taken from a palp after WMFISH utilizing a digoxigenin‐labelled riboprobe of OBP1.
