# Supplementary material for: Expression of odorant‐binding proteins in mouthpart palps of the desert locust Schistocerca gregaria
Source: Insect Mol Biol. 2018 Nov 27;28(2):264–76. doi: 10.1111/imb.12548 (PMC7380039; doi:10.1111/imb.12548)
Supplement: Supplementary file 1 — Figure S1 . (A–C) FISH on section of labial (A and B) and maxillary (C) palps using biotin‐labeled riboprobes of subfamily‐IA OBP1, OBP5 and OBP6. The white dash‐line indicates the boundary to the cuticle. (A′–C′) Magnification of the boxed area in A‐B (white dash‐line). Images represent projections of different optical layers from confocal image stacks or are a single confocal image from an image stack. Scale bars: A–C 50 μm, A′ 20 μM and B and C′ 10 μm. Figure S2 . (A–C) FISH on maxillary (A, B and D) and labial (C) palps of Schistocerca gregaria with digoxigenin‐ and biotin‐labeled riboprobes of OBP2, OBP4, OBP7 and OBP8. The white dash‐line indicates the boundary to the cuticle. Images represent projections of different optical layers from confocal image stacks or are a single confocal image from an image stack. Scale bars: A–D 50 μm. [file IMB-28-264-s001.pdf]

Supplementary material

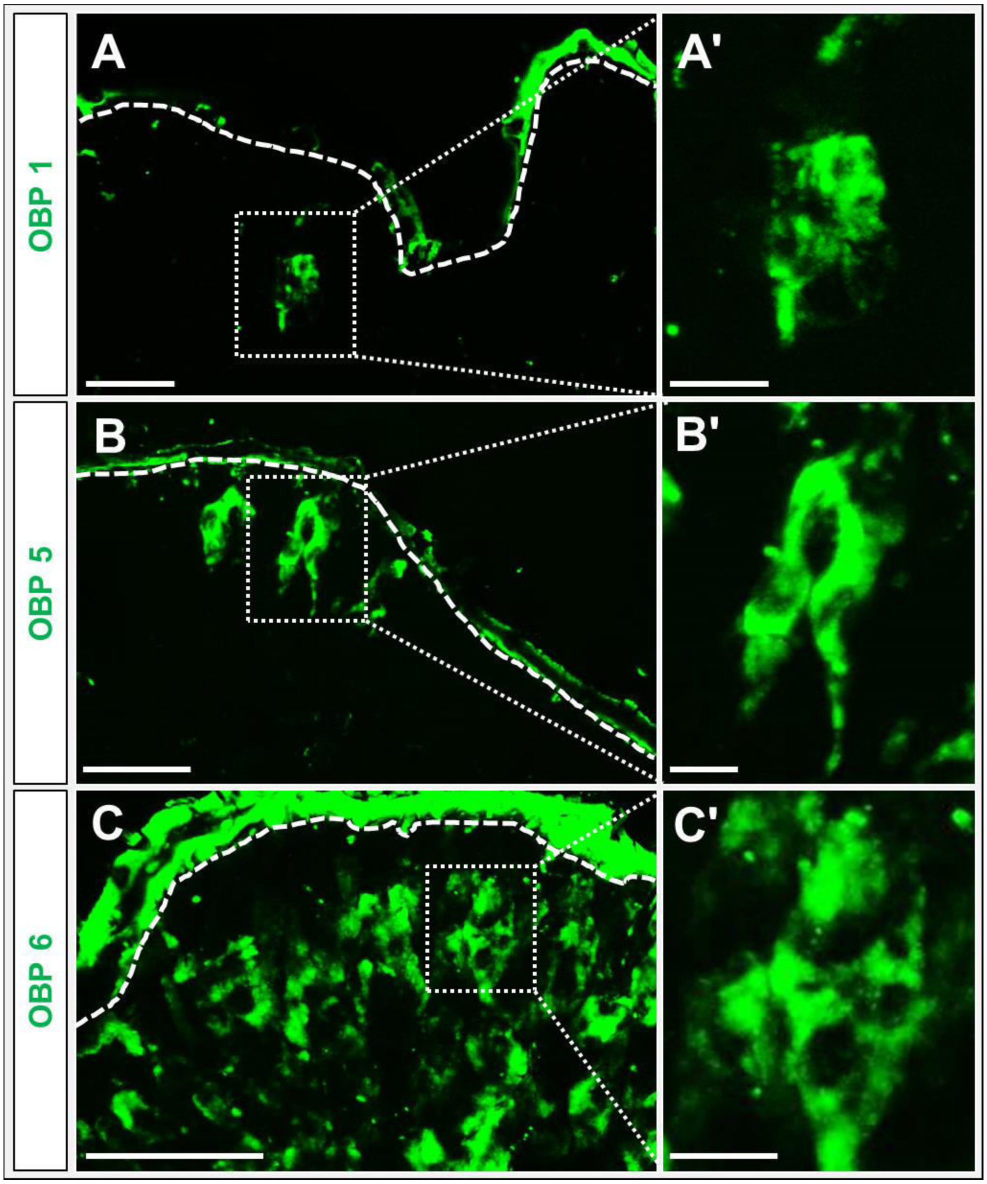

Fig. S1

(**A-C**) FISH on section of labial (A-B) and maxillary (C) palps using biotin-labeled riboprobes of subfamily-IA OBP1, OBP5 and OBP6. The white dash-line indicates the boundary to the cuticle. (**A'-C'**) Magnification of the boxed area in A-B (white dash-line). Images represent projections of different optical layers from confocal image stacks or are a single confocal image from an image stack. Scale bars: A-C 50 µm, A' 20 µM and B-C' 10 µm.

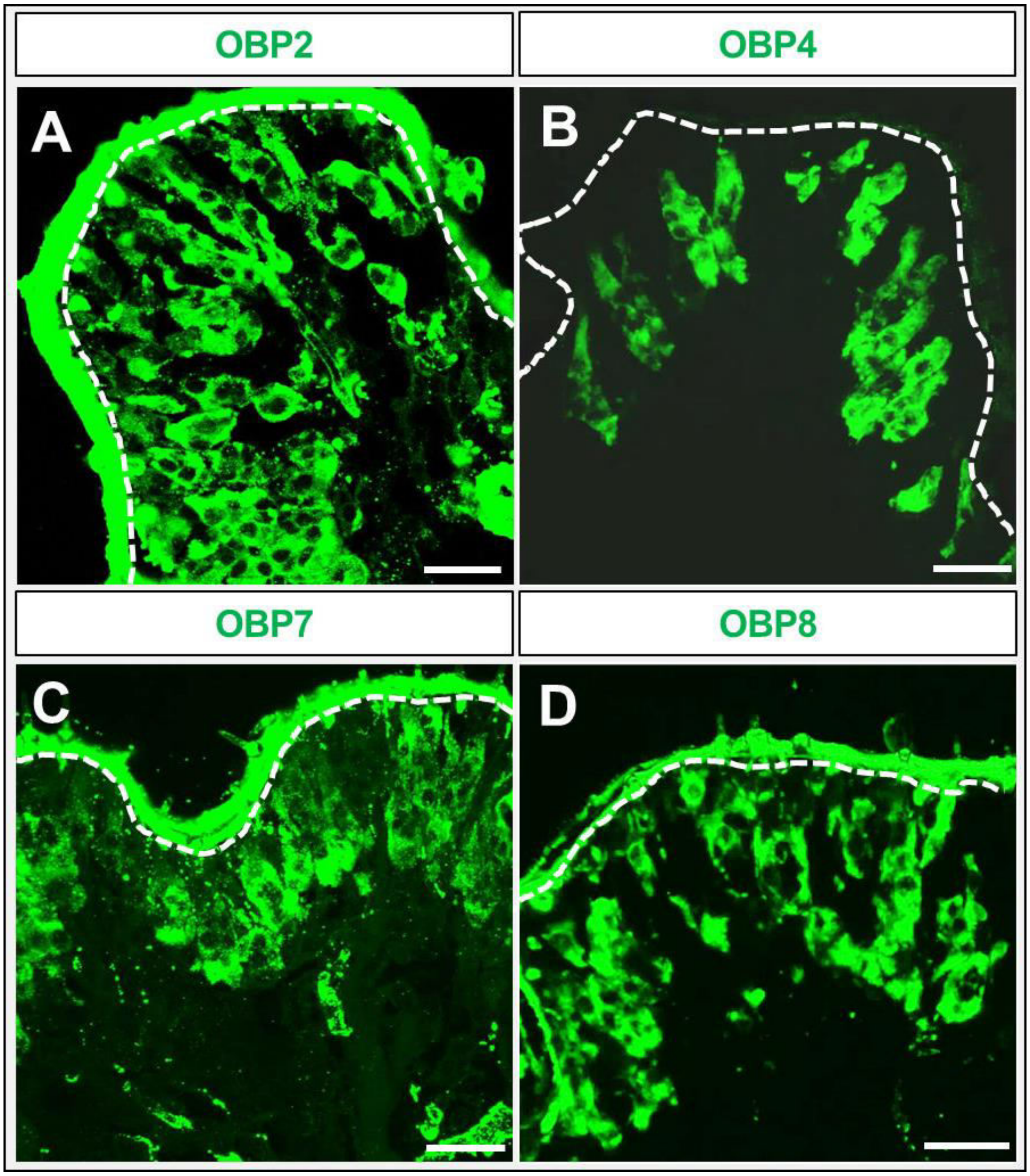

Fig. S2

(A-C) FISH on maxillary (A-B and D) and labial (C) palps of *Schistocerca gregaria* with digoxigenin- and biotin-labeled riboprobes of OBP2, OBP4, OBP7 and OBP8. The white dash-line indicates the boundary to the cuticle. Images represent projections of different optical layers from confocal image stacks or are a single confocal image from an image stack. Scale bars: A-D 50 µm.

Video S1

A video generated from single plane confocal images of taken from a palp after WM-FISH utilizing a digoxigenin-labelled riboprobe of OBP1.
